# Lasso and XGBoost‐Enabled Prediction Models for Sensory Dysfunction, Biological Age, and APOE Genotype in Cognitive Decline Risk Assessment

**DOI:** 10.1002/alz70856_107762

**Published:** 2026-01-09

**Authors:** Longjian Liu, Jintong Hou, For the CKM‐AD/ADRD Risk Prediction Study Group

**Affiliations:** ^1^ Drexel University Dornsife School of Public Health, Philadelphia, PA, USA

## Abstract

**Background:**

Cognitive decline is a major public health concern, with growing evidence linking sensory dysfunction, biological age, and APOE genotype to an increased risk of neurodegenerative disorders, including Alzheimer's Disease (AD) and AD‐related dementia (AD/ADRD). Advanced machine learning methods, such as Least Absolute Shrinkage and Selection Operator (Lasso) regression and Extreme Gradient Boosting (XGBoost), offer robust predictive capabilities for identifying high‐risk individuals. This study leverages data from the Health and Retirement Study (HRS) to develop and validate predictive models integrating these key factors.

**Methods:**

We analyzed a nationally representative cohort of 13,222 U.S. adults aged ≥50 years from the 2008 HRS dataset. Sensory function was assessed via measures of vision, hearing, and taste impairment. Biological age, a novel index, was derived from clinical biomarkers associated with cardiovascular, kidney, and metabolic (CKM) disorders—including hypertension, systolic blood pressure, serum cystatin C, and hemoglobin A1c—alongside chronological age. Cognitive function was evaluated using a validated 27‐point scale, with cognitive decline defined as the lowest quartile (Q1). APOE alleles were genotyped to assess genetic risk. Lasso regression was applied for feature selection, while XGBoost was used to develop high‐performance prediction models. Model performance was evaluated using cross‐validation and assessed through the area under the receiver operating characteristic curve (AUC‐ROC), sensitivity, and specificity metrics.

**Results:**

Among 13,222 participants, 7.6% experienced visual decline, 23.3% had moderate to severe hearing decline, and 4.9% reported loss of appetite. Sensory impairments (vision, hearing, and taste loss) and biological age were significant contributors to cognitive decline risk, with APOE‐ε4 carriers exhibiting the highest susceptibility. Lasso regression and XGBoost outperformed traditional logistic regression models in classification accuracy. The inclusion of sensory function measures significantly enhanced predictive performance (AUC‐ROC >73%).

**Conclusion:**

Our study demonstrates the utility of machine learning‐based models in cognitive decline risk assessment, emphasizing the predictive value of sensory dysfunction, biological age, and APOE genotype. These findings underscore the importance of early screening and targeted interventions for at‐risk populations. Future research should explore model generalizability across diverse cohorts and incorporate additional biomarkers to enhance precision and clinical applicability.

